# Computational Assessment of Neural Probe and Brain Tissue Interface under Transient Motion

**DOI:** 10.3390/bios6020027

**Published:** 2016-06-16

**Authors:** Michael Polanco, Sebastian Bawab, Hargsoon Yoon

**Affiliations:** 1 Department of Mechanical and Aerospace Engineering, Old Dominion University, Norfolk, VA 23529, USA; mpola002@odu.edu (M.P.); sbawab@odu.edu (S.B.); 2 Department of Engineering, Center for Materials Research, Norfolk State University, Norfolk, VA 23504, USA

**Keywords:** neural electrodes, brain, finite element analysis, viscoelasticity

## Abstract

The functional longevity of a neural probe is dependent upon its ability to minimize injury risk during the insertion and recording period *in vivo*, which could be related to motion-related strain between the probe and surrounding tissue. A series of finite element analyses was conducted to study the extent of the strain induced within the brain in an area around a neural probe. This study focuses on the transient behavior of neural probe and brain tissue interface with a viscoelastic model. Different stages of the interface from initial insertion of neural probe to full bonding of the probe by astro-glial sheath formation are simulated utilizing analytical tools to investigate the effects of relative motion between the neural probe and the brain while friction coefficients and kinematic frequencies are varied. The analyses can provide an in-depth look at the quantitative benefits behind using soft materials for neural probes.

## 1. Introduction

Various neural probes have been developed for neuro-chemical and electrical signal sensing inside the brain [1,2,3,4,5,6,7,8,9]. By using chronically implanted probing devices, single neural cell and/or multi-neural ensemble recording could be available over a long period of time. Efforts continue to enhance the yield and longevity of chronic neural recordings in laboratory animals. Even with successful implementation of neural sensing devices in the brain, it is important to address longevity issues in chronic neural recording. The recording capabilities of microelectrodes have been mostly limited to a few months; they then degrade over time due in part to neuronal cell degeneration and cell layer formation on the electrodes from the inflammatory immune response caused by the chronic implantation and operation [10,11]. It is reported that the inflammatory response to mechanical impact also gives rise to Interleukin-1β, a proinflammatory cytokine derived from the caspase-1 enzyme [12,13]. Recent studies have shown a promising result for pharmacologic intervention that proves that the recording capabilities of electrodes can be improved by blocking caspase-1 activity [14].

To mitigate cellular and molecular response to the implanted probe, various materials and designs of neural probes and the local delivery of anti-inflammatory drug have been investigated to enhance electrode longevity [15,16,17,18,19]. Some other approaches such as nerve growth factor application or rejuvenation process using electrical pulses may be another useful strategy to prolong the lifetime of chronically implanted microelectrodes [20,21,22]. Many researchers have focused on using soft materials to mediate tissue and cell damage from mechanical motion in the brain [18,19].

While silicon and metal probes have traditionally been utilized for neural sensing applications, soft polymer materials may have an advantage due to their greater flexibility and biocompatibility [18,23,24]. One major problem that occurs when interfacing neural probes and brain tissue is related to the large difference in Young’s Modulus (10^5^–10^7^ Pa) between them [25]. During chronic recording, the brain is subjected to movement brought upon by external motion of the body due to cardiac pulse, breathing, rapid head movement or impact collision. The difference in material stiffness between the brain and the probe makes the brain microvasculature and neural cells more susceptible to mechanical damage such as glial scarring. To mitigate these damage mechanisms, it is advantageous to design a probe that will minimize damage to the brain by limiting relative motion between the embedded neural probe and brain tissue, and ultimately enhance the quality of neural sensing and functional lifetime of the probe.

There have been several research efforts to implement materials with stiffness values close to that of brain tissue [26,27,28,29,30,31]. In Kim’s research, hydrogel coatings were applied to surface of neural electrodes for better integration and mechanical buffering between the electrodes and neural tissue. Kipke’s group tested Polydimethylsiloxane (PDMS) probes in an agarose *in vitro* brain model using stiff silicon insertion shuttles that can resolve a conflicting issue of precise implantations and mechanical softness. A composite neural probe with Parylene-C and polyethylene glycol (PEG) with integrated microfluidic channels was investigated. In this approach, PEG inside the microchannels makes the probe stiff and allows for precise positioning of the probe into a cortex. The PEG dissolves upon insertion into the brain resulting in a mechanically soft structure. Work continues in this area to quantify the mechanical compliance of softer probes for coupling with the brain.

To aid in increasing longevity of neural probes, previous research using finite element analysis has addressed the effects that micromotion can have on the brain when an embedded neural probe is present using different coatings and probe material properties [12,32,33,34,35]. Studies have also been conducted examining the brain tissue over a period of weeks using embedded probes of varying stiffnesses [36]. However, the analyses have been largely limited to static deformation, and limited research has been performed to understand the effect of transient motion, or frequency, on the brain. The effects of transient motion have been studied by Zhang [37], who only looked at the brain’s response to a silicon probe during the motion. Time history displacement test data was acquired from Differential Variable Reluctance Transducers (DVRT) that were placed on the surface of anesthetized rat brains [38,39]. The displacements recorded in the brain due to respiration can range between 2 and 25 µm while the frequencies corresponding to those displacements are approximately 1–2 Hz [38,39]. Likewise, the displacements corresponding to cardiac pulse are slightly lower at 1–4 µm [38,39] with frequencies reaching up to 5 Hz. These measurements were taken with the brain exposed and a portion of the skull removed.

The major interest of this study is to investigate the transient motion of the brain with frequencies between 1 and 40 Hz applying mechanically-compliant materials for a neural probe and illustrate the post-insertion behavior of the probes during head movements that are likely to produce significant micromotion between the probes and surrounding brain tissue. While rat physiology will be employed in the studies, the frequencies are chosen such that practical applicability to humans can be extrapolated. For example, the frequencies in humans that can be generated within the head during walking can reach approximately 24 Hz at resonance [40]. Previously, transient frequencies at 1 and 5 Hz and Young’s Moduli ranging from 165 GPa (silicon) to 6 MPa were considered [41]. The latter was driven by a finding shown in Lee [33] showing silicon microelectrodes to be capable of producing strains up to approximately 25% within the brain under a 1 µm longitudinal static displacement during initial insertion, or no coupling between the probe and the brain. The present study, using Finite Element Analysis techniques, covers a full range of friction coefficients to simulate different events in the probe implantation period from initial insertion to bonding, as represented by the formation of microglia/microphages around the implant site.

The intention of this study is to capture the interaction of the probe onto the brain due to relative micromotion. The configuration assumes that the probe being fixed to the skull complies with conventional animal experimental conditions using depth probes. It is expected that the relative motion between the probe and the brain still occurs [42] despite being enclosed in the skull where micromotion may be more inhibited. The outcome of this configuration can be applied and useful for future analysis of floating electrodes on the cortical surface including the cerebrospinal fluid within a hydrostatically sealed cranial structure. Since the state-of-the-art is gravitating towards manufacturing mechanically compliant neural probes to increase its longevity, a low modulus value of 200 kPa resembling hydrogel-type materials is studied and compared with a traditionally used silicon probe in the assessment of mechanical mismatch reduction between the probe and the brain.

## 2. Materials and Methods

The same quarter-symmetry finite element model (FEM) and boundary conditions described in [41] were utilized for this study. Quarter-symmetry boundary conditions assume that, in a full-scale representation, all kinematic motion and behavior from the interaction between the probe and the brain is mirrored along all symmetry planes, and has been utilized in previous studies [33,34,36,37]. The probe dimensions for the Michigan electrode were taken for our study and are described in [33], which represents a typical geometric representation of a silicon microelectrode. The probe was assumed to be placed in the center of a full brain model. Accordingly, the dimensions of the probe for the quarter-symmetry model were 7.5 µm thick, 60 µm wide, and 3000 µm long (note that both the half thickness and half widths were modeled in accordance with quarter-symmetry dimensions). Since microelectrodes can record from neurons within a radius of approximately 140 µm radius from the implant site [43], and damage due to micromotion could occur in cells within a 60 µm radius [25], the dimensions were chosen to be much greater than 140 µm to sufficiently capture deformation patterns within the brain. Thus, the dimensions of the brain model are 750 µm wide, 750 µm thick and 5000 µm long. The model contained a total of 11,860 elements and 14,794 nodes. A mesh bias was applied to concentrate the higher element density towards the interface between the brain and the probe, where the maximum deformation was expected to occur.

The nonlinear transient finite element code LS-DYNA [44] Version 971 R6.0 was utilized since the nature of the micromotion profiles was transient. A reduced integration formulation for solid elements [45] in LS-DYNA was utilized to characterize elements in both the probe and the brain. Using traditional contact definitions [46] within the software, three coefficients of friction were chosen for the simulations: 0.3, 0.6, and 1. The coefficients were chosen to simulate different periods of time for the probe to brain interface. A coefficient of friction of 0.3 signifies a period of initial insertion, or no coupling, a coefficient of 0.6 simulates increased, yet loose, bonding of the probe to the brain due to microglia formation, while a coefficient of 1 represents full bonding denoted by the microglia formation.

An elastic formulation on the probe was employed for the 200 kPa Young’s Modulus similar to the property of some hydrogels (v = 0.33), while a rigid formulation was employed for the silicon probe to prevent allow for quicker run times in LS-DYNA (E = 165 GPa, v = 0.22). These property values for silicon were the same values utilized in Lee [33]. A literature survey on properties conducted by Yang [47] highlights the various methods researchers have used to characterize the brain, including hyperelastic [48], elastic, and viscoelastic [49,50,51,52] formulations, sometimes with user‑defined material models [52,53]. For our application, a linear viscoelastic material model with properties acquired from [54] for head impact studies was used to characterize the brain since small displacements on the order of tens of micrometers were included in our modeling. The material properties utilized for the brain can be found in Table 1. The shear behavior for a linear viscoelastic model is characterized using the following constitutive equation as a function of time: G(t) = G_∞_ + (G_o_ − G_∞_)e^−βt^, where β is the decay constant, while being elastic in compression. The bulk modulus utilized here has been derived from testing [55] and has been commonly used for brain injury research. The shear properties utilized for this study account for the stiffness in the brain due to ventricles, and, as stated by [56], the ratio between bulk and shear moduli is approximately 10^5^, to define the incompressible nature of the brain and its low stiffness in shear. As the intent of this study is to capture the interaction of the probe onto the brain solely due to relative micromotion, cerebrospinal fluid was absent from the analyses at this time for simplicity and comparative analysis with previous studies.

The top surface of the probe was fixed to represent an attachment of the neural probe to the skull. All input pulses were applied to the bottom surface of the brain in accordance with micromotion propagation onto the probe. The frequencies chosen and applied in the analyses, ranging from 1 Hz to 40 Hz, are most pertinent to the transient motion brought upon by rigorous motion that has the neural probe embedded in their brain. For this study, a low magnitude displacement of 4 µm was chosen to cover the 1–40 Hz frequency range being considered for the transient analyses. Only longitudinal motion was considered for the analyses since it has been shown to inflict greater injury by the probe during mechanical motion [33,34,37]. To assess the behavior of the brain at higher frequencies over time, the cases were run for three cycles using the 4 µm magnitude using a less stiff probe (200 kPa) over the 1–40 Hz frequency range. Due to long run times as a result of higher moduli associated with the probe, analyses using the silicon probe were relegated to only one cycle of motion. The intent was to capture the deformation of the brain at peak displacement after the probe has been inserted into the brain, as maximum strain was expected to take place at the initial period of displacement such as the onset of head impact.

## 3. Results

Both Von Mises stress and strain were examined in the analyses to take into account the combined compression-extension and shearing that takes place along the probe interface during brain motion. Stress and strain values were acquired for FEM elements that are adjacent to the interface, expecting that the deformation would be greatest in this region of the brain. Table 2 and Table 3 show the side stresses and strains at the tip of the probe-brain interface at maximum displacement of the brain for the first motion cycle using various friction coefficients for both probes. The stresses increase monotonically with the applied displacement frequency by over 1000 Pa. Values in the tables show two things: differences in stress and strain values over various degrees of probe/brain coupling, and peak deformation values converge as bonding increases along the interface. In addition, the stress level is kept relatively steady and small compared to silicon when Young’s Modulus is reduced to 200 kPa over the frequency range of 1 to 40 Hz. Over lower frequencies, the softer probe exhibits high stress and strain levels compared to the characteristics at higher frequencies due to a conversion of brain kinetic energy to strain energy in the probe. Around 20–30 Hz, however, a slight increase is seen due to an increase in impulsive effects between the probe and the brain, though the levels never reach as high as those seen around 1–5 Hz. The relative reduction in stress and strain compared to silicon does not go below approximately 95% over the 1–40 Hz frequency range compared to silicon.

**Figure 1:**
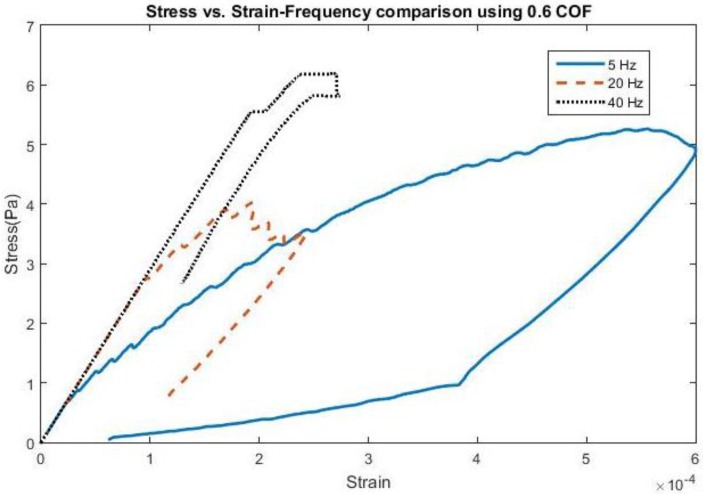
Stress *vs.* Strain for a 4-micron amplitude at various applied frequencies using a coefficient of friction (COF) of 0.6.

**Figure 2:**
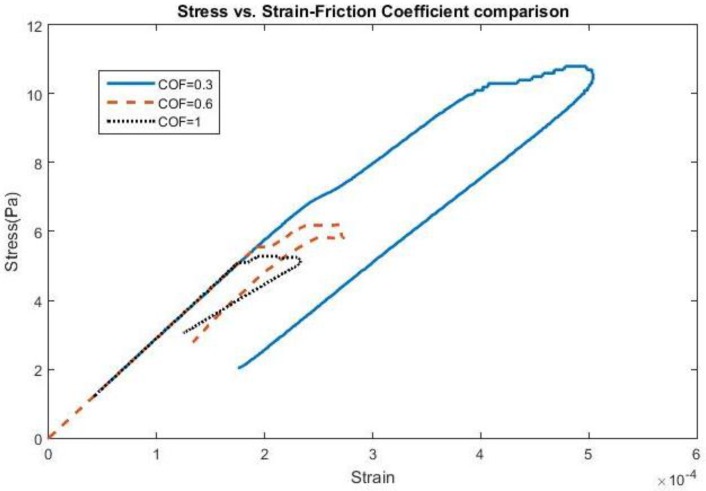
Stress *vs.* Strain for a 4-micron amplitude at 40 Hz using various COFs.

**Figure 3:**
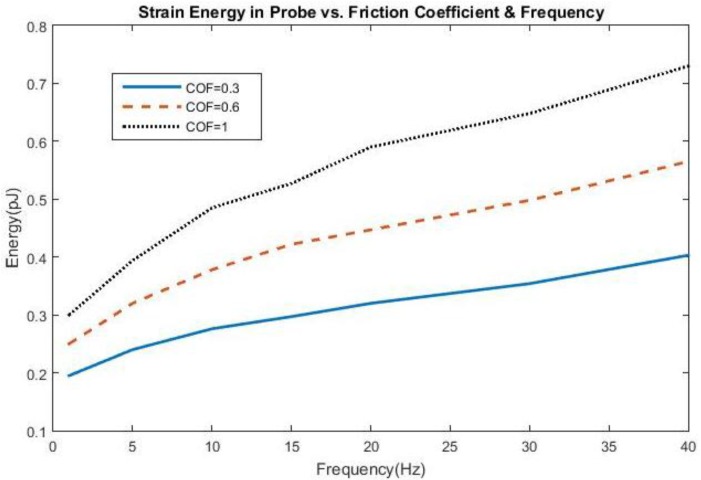
Strain Energy in the 200 kPa probe as a function of friction coefficient and frequency.

The effect of frequency change and adhesion on stress and strain can be found in Figure 1 and Figure 2, all shown for the first cycle of motion for the 200 kPa probe. The hysteresis loop, shown for a friction coefficient of 0.6, encompasses a wider range of stress and strain at lower frequencies due to a longer time of contact between the probe and the brain during the first cycle and returning close to its original state. As the frequency increases to 20 Hz, the stress levels decrease compared with 5 Hz, possibly due to strain energy being absorbed by the soft probe as a result of the kinetic energy increase. Sinusoidal effects are present due to the increased presence of friction along the probe-brain interface. However, when the frequency reaches 40 Hz, the stress levels increase again, possibly due to inertial effects being present, leading to increased indentation with larger kinetic energies present. The strain levels also end at the same values before undergoing another cycle of motion. A low friction coefficient (COF = 0.3) unsurprisingly exhibits high stresses and strains before settling at a strain value during the first cycle. However, once the friction coefficient reaches 0.6, the stress and strain behavior is nearly similar to that with a friction coefficient of 1, or total bonding. This behavior suggests similarity with other degrees of bonding once it has been achieved. The strain, or deformation, energy in the 200 kPa probe is shown in Figure 3. The energy increases directly both with friction, or the degree of interface bonding, and frequency in the brain as work done by friction acts to resist the motion in the brain in creating increased deformation. The silicon probe is not shown as the strain energy was shown to be negligible due to its relatively high stiffness.

**Figure 4:**
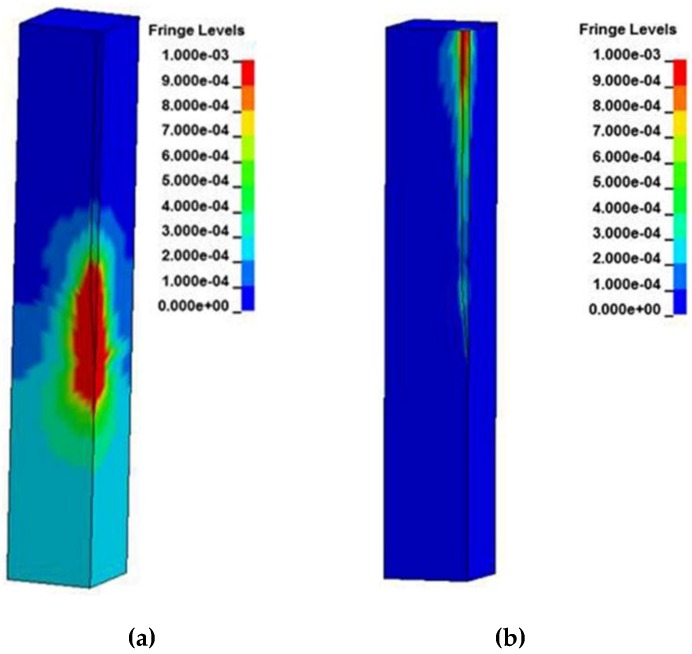
Strain distribution of the brain using a micromotion displacement of 4 µm and interfaced with a (**a**) silicon probe and (**b**) 200 kPa stiff probe.

Fringe plots for the brain at peak strain levels can be found in Figure 4 for the 20 Hz applied displacement frequency. The strain levels scale goes to 0.001 while the stress levels scale goes to 1 × 10^−7^ Pa. This is not a reflection of the peak stresses and strains that occur during the duration of the displacement cycle, but was chosen to show how the distributions are distributed at peak displacement. The deformation profiles when the Young’s Moduli of silicon and 200 kPa are employed are shown. As the Young’s Modulus of the probe decreases, the deformations of the brain also decrease around the tip region and do not spread out as far from the interface, eventually showing negligible deformation around the tip when Young’s Modulus is 200 kPa. It is noted also that the stresses and strains exhibit linear behavior until reaching the taper at position 2.3 mm, where both compression and shear deformations occur and exhibit their highest values. Sinusoidal effects are present in the traces due to the coupled role of friction and probe strain energy during motion of the brain.

**Figure 5:**
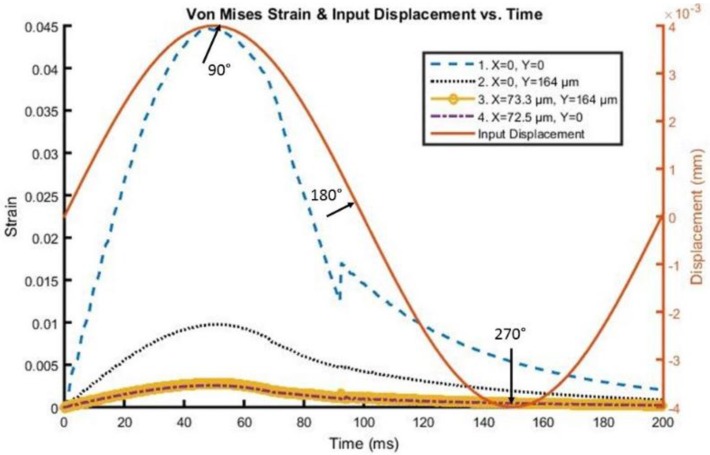
Von Mises Strain *versus* time along the interface of the Silicon probe with the brain.

**Figure 6:**
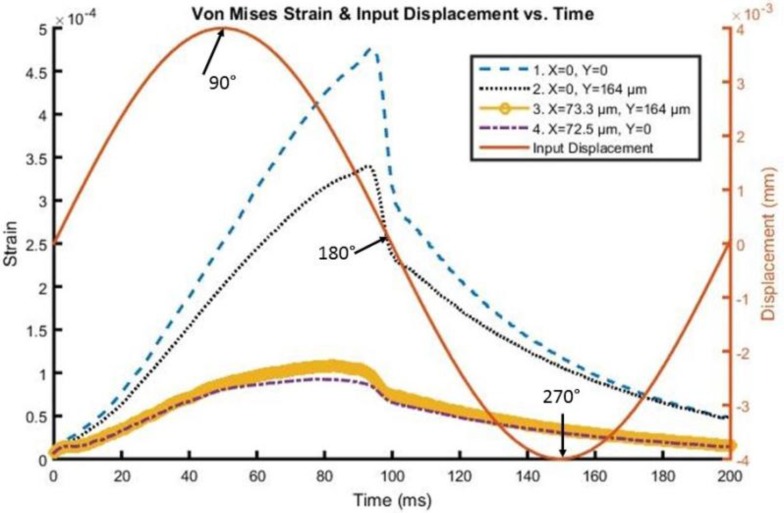
Von Mises Strain *versus* time along the interface of the 200 kPa stiff probe with the brain.

**Figure 7:**
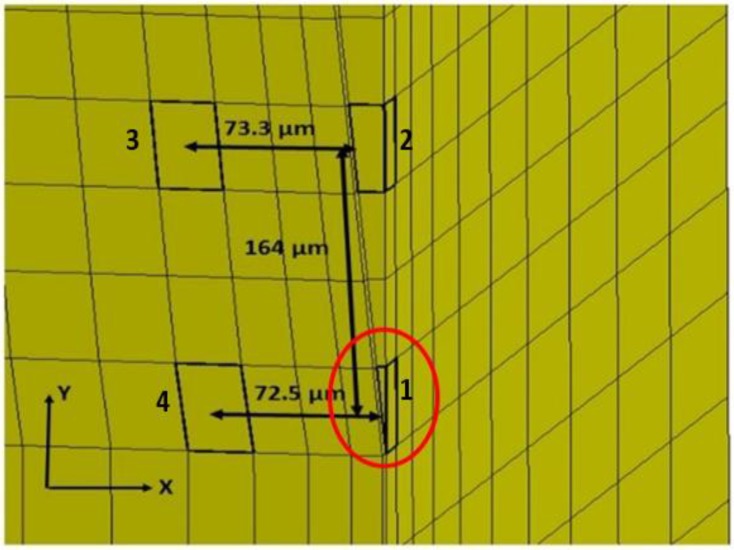
Element locations along interface tip for time history traces corresponding to graphs in Figure 5 and Figure 6.

A time history of strain element response in the tip region, as far away as approximately 73 microns from the interface and 164 microns from the actual tip of the probe, can be found in Figure 5 and Figure 6 for both silicon and the probe with 200 kPa Young’s Modulus at 5 Hz and 4 microns to depict the brain response, or deformation levels from the probe, during vascular pulsation. Each output is compared with the input micromotion profile. The elements graphed are highlighted in Figure 7. The graphs show that the highest strain level occurs near the interface where the probe tip is located (denoted in red as x = 0 and y = 0) and decreases the farther away one gets from the interface. The locations plotted in Figure 4 and Figure 5 are denoted by numbers 1–4 in Figure 6. The strain level is highest at approximately 50 ms, when the pulse reaches its amplitude before decreasing. A discontinuity is shown at approximately 90 ms within the simulation for a silicon probe in Figure 4, which signifies that the probe is still in contact with the brain for a short duration as it changes direction in motion. The discontinuity is not seen in the graphs; however, for the softer probe (200 kPa), indicating that the probe is soft enough to absorb the energy from brain micromotion and would not result in contact with the tip for extended periods of time. Compared with use of the silicon probe, the brain experiences a time lag in peak deformation using a softer probe due to energy absorption within the probe during motion. As the silicon probe deformation is negligible, peak deformations in the brain do not lag with respect to time.

**Figure 8:**
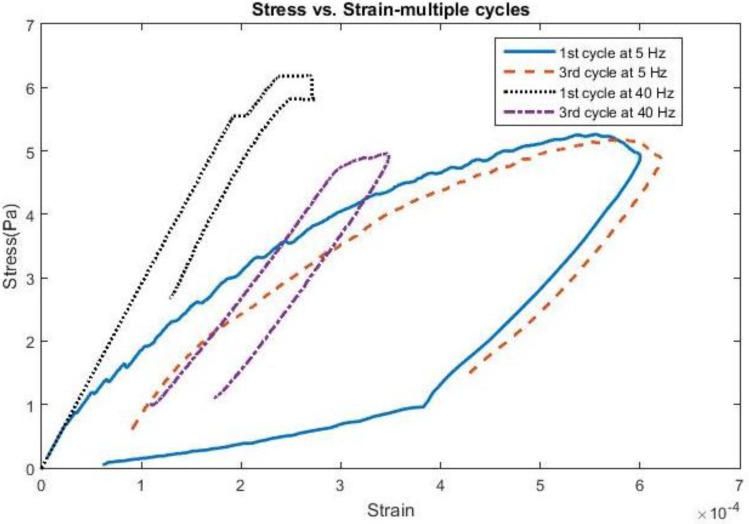
Stress *vs.* Strain for 4 microns comparing first 3 cycles of motion using 200 kPa Young’s Modulus.

Finally, examples of stress and strain behavior over multiple cycles of motion can be seen for a 200 kPa probe in Figure 8 for a 4 µm displacement pulse at both 5 Hz and 40 Hz for a COF of 0.6. At 5 Hz, the stress and strain behavior is almost identical between the initial and third cycles, indicating that the kinetic energy from the brain is not sufficient to produce deformations higher than its initial value. At 40 Hz, the stress levels are higher, but also the strains are not as high as they are at lower frequencies due to the strain energy of the probe becoming more prevalent with the increase of the brain’s kinetic energy. The traces also indicate the role that strain relaxation time plays between indentation cycles and their contribution to the strain experienced within the brain.

## 4. Discussion

Using state-of-the-art tools in Finite Element Analysis, an examination of the behavior of structures under various loading conditions can be performed, providing insight on what parameters are important when designing neural probes. For this problem, researchers have been looking at ways to reduce the strains seen at the tip of the probe along the brain tissue interface as a result of mechanical mismatch between the neural probe and brain tissue. The results presented attempt to provide guidance on what is needed to avoid injury to the brain around the probe tip resulting from mechanical motion of the brain. To the authors’ knowledge, the transient aspects of motion within the brain utilizing materials for the probe compatible to the brain had not yet been addressed, and, in conjunction, the benefit probes with lower Young’s Moduli play in providing strain relief under different motion profiles was investigated in this study. Through incorporation of frequency, the study offers a more realistic approach to examining the probe to brain interface over time due to micromotion.

The aim of the analysis was to show the motion in the first few moments after the onset of mechanical impact during various stages of probe insertion. The brain tissue was presumed to be more vulnerable to the mechanical damage during the initial period of interface. By utilizing probes closely resembling mechanical characteristics of the brain, the deformation levels can be reduced as highlighted by the scenarios in this study. A Young’s Modulus of 200 kPa was examined as it was hypothesized that strain levels at the tip region of the brain could be alleviated. These values for the probe were tried in several research efforts by implementing hydrogel materials [27,28]. Over time, while the strain levels may continuously build up, in any micromotion profile, they are considered miniscule enough using softer probes that they likely would not cause any injury to surrounding tissue, as evidenced by the hysteresis plots presented. A limitation of the study included run times that ranged between eight hours to over a day depending on the frequency employed to run the models on four CPUs. In addition, while some of the micromotion profiles applied were based on previously conducted rat experiments [34,35,38,39], overall the analyses provide an approximation on the implications movement from pulsation, breathing, and walking can have on a human patient.

Introducing different friction coefficients to simulate bonding between the probe and the brain also provides insight as to how the brain is affected over the period of implantation with respect to frequency and Young’s Modulus. In the event that the brain undergoes rigorous motion due to walking or head movement, more energy can be absorbed by the probe as the degree of bonding increases over time due to better material compatibility. In addition, the simulations suggest that once bonding is introduced, the strain levels, or potential for scarring, decrease significantly compared with scenarios where the probe has initially been implanted into the brain and the damage to tissue is more imminent. However, as astro-glial sheath formation can occur as early as approximately 2 to 4 weeks after initial insertion into the brain [57], the probe’s ability to record neuronal activity can diminish from separation between neural spike sources and sensing electrodes on the probe [58,59]. To facilitate bonding to the brain during implantation, a coating around the probe can help in alleviating deformation levels as suggested in Lee and Subbaroyan [33,34].

It should be noted that a linear viscoelastic formulation was utilized to characterize the brain in this study. Previous research investigated the effects of micromotion [33,34] using small displacements (1–4 µm) in the static regime, accordingly characterizing the brain using an elastic formulation. However, when dealing with variable strain rates and transient, or impact, motion, it is most appropriate to characterize the brain using either a viscoelastic or hyperelastic formulation to effectively capture brain deformation under different micromotion profiles primarily due to shearing from the probe.

The analyses presented provide an initial step in validating the interfacial effects between the probe and the brain in accordance with physics due to relative brain motion. More in-depth examinations on the effects of cerebrospinal fluid on interaction between the probe and the brain can be conducted in the future as it is expected to dampen motion when the fluid fills the voids within the brain. Moreover, application of a lower probe Young’s Modulus in the analyses serves as a precursor to wider applications in neural probe design. Future studies can be extended to include transient models incorporating stiffer probes integrated with softer materials such as Silk, PEG, or Polydimethylsiloxane (PDMS), as a surface coating layer of probe or a bioresorbable backing structure. Using a viscoelastic model for the brain and exploring material alternatives for neural probes provides insight for a next generation of neural interface technology. In the future, boundary conditions can be modified to model an untethered probe in the cerebral cortex for chronic recordings in human and large animal applications in an air-tight seal skull condition, which incorporates a dominant tangential component of deformation, or an electrode tethered to the skull by way of a flexible cable [42].

## 5. Conclusions

A series of transient brain motion profiles were employed and the assessment of mechanically compliant neural probes examined at the interface between the probe and brain tissue was completed. The frequency was varied between 1 and 40 Hz to determine the behavior of the rat brain under mechanical motion as a result of various motor functions such as vascular pulsation, respiration, and walking. The cases were run to three cycles of motion using a probe Young’s Modulus of 200 kPa as running multiple cycles resulted in negligible change in stress and strain. Deformation levels in the brain start converging when bonding becomes more present along the interface, as simulated with higher friction coefficients. The stress-strain profiles also differed vastly between motion cycles as frequencies increased while low deformation levels were maintained. By applying transient pulses, the interaction between the probe and the brain as the kinetic energy varies was examined, and revealed the importance of strain relaxation within the brain on deformation levels between indentation cycles. Furthermore, at least a 95% reduction in stress and strain was present at the tip when a 200 kPa Young’s Modulus was utilized for the probe, highlighting the importance of using probes mechanically similar to that of the brain to reduce long-term injury to surrounding tissue.

## Figures and Tables

**Table 1:** Brain viscoelastic material properties.

	Density (kg/mm^3^)	Bulk Modulus (GPa)	Short-term Shear Modulus, G_o_ (kPa)	Long-term Shear Modulus, G_∞_ (kPa)	Decay constant, β (m·s^−1^)
Brain	1.05 × 10^−6^	2.1	10	2	0.08

**Table 2:** Maximum stress side interface values for 165 GPa (silicon)/0.0002 GPa probe at the tip.

Frequency	Interface Stress (Pa) with 0.3 COF	Interface Stress (Pa) with 0.6 COF	Interface Stress (Pa) with 1 COF
1 Hz	325/14.7	304/6.62	291/3.92
5 Hz	607/12.9	570/5.27	545/2.92
10 Hz	857.5/11.6	813/3.7	764/2.96
15 Hz	1010/10	955/3.38	906/3.46
20 Hz	1149/9.3	1080/4.02	1011/3.67
30 Hz	1310/10	1230/5.06	1146/4.79
40 Hz	1400/10.8	1350/6.2	1260/5.53

**Table 3:** Maximum strain side interface values for 165 GPa (silicon)/0.0002 GPa probe at the tip.

Frequency	Side Interface Strain with 0.3 COF	Side Interface Strain with 0.6 COF	Side Interface Strain with 1 COF
1 Hz	0.0503/2.14e−3	0.0474/1.03e−3	0.0448/5.67e−4
5 Hz	0.052/1.45e−3	0.049/6e−4	0.0463/3.29e−4
10 Hz	0.0529/9.38e−4	0.0497/3.27e−4	0.0472/2.3e−4
15 Hz	0.0535/7.11e−4	0.05/2.58e−4	0.0478/2.08e−4
20 Hz	0.0539/5.91e−4	0.0511/2.42e−4	0.0487/1.94e−4
30 Hz	0.054/5.24e−4	0.0514/2.42e−4	0.0487/2.06e−4
40 Hz	0.0583/5.05e−4	0.0552/2.74e−4	0.0515/2.33e−4
